# The Radium Fetich

**Published:** 1916-09

**Authors:** 


### The Radium Fetich.

Anything which is mysterious seems to appeal to such an alarming proportion of people. And when one can place such a potent mystery in so small a package as a radium capsule the mystery deepens.

The refinement of radium reduction from huge masses of ore permits the physician to secure 25, 50 or 100 milligrams of radium element in a small glass capsule which is measured by millimeters. In fact, 50 milligrams of radium element would just about equal the amount of fuller's earth that one would place upon the point of a pocket-knife blade. Yet this almost insignificant amount of matter can produce remarkable changes in tissues, especially malignant tissue. Is it any wonder that such pygmian potency appeals to the plebeian love for mystery?

Therapeutically, there are two principal ways of applying radium. For skin diseases radium plaques or squares of lead are varnished upon one side with a radium salt. These are made in various sizes and with any amount of radium desired. They are from one to three square inches in size and usually contain from 10 to 20 milligrams of radium element. These are generally used only for superficial skin treatment. They are of little, if any, value in deep cancer.

The other principal method is radium in capsules. These can be obtained in various sizes containing 10 to 100 milligrams. It is generally considered that no capsule containing less than 25 milligrams of pure radium element will be useful in deep therapeutics. A 50-milligram capsule is better and far more commendable.

Of course, the ideal method of pursuing radium therapy is by emanations; but this is not practical unless 300 to 500 milligrams of the element is available. Producing radium emanations is a problem for the physicist rather than the physician. This method is quite complicated and will be described at another time. It is to be remembered that nowadays all radium is bought and sold upon its percentage of pure radium element. It is marketed, of course, not in the pure element but as the bromide sulphate, etc. Purchase measurements are made at so much per milligram of pure radium element, however. The old method of measuring by the activity of the radium salt is obsolete. All radium can now be measured by the United States Bureau of Weights and Standards, and such standardization should be demanded by any purchaser. There is no reason why anyone should be disappointed in radium purchases in America with such good government standardization. E. H. S.

---

"How's the patient this morning?"

"The doctor says he's got potamaine poisoning and is in a catamose condition."—*Alice Echo.*

---

### Do You Know That

Intelligent motherhood conserves the nation's best crop?

Heavy eating like heavy drinking shortens life?

The registration of sickness is even more important than the registration of deaths?

The U. S. Public Health Service co-operates with State and local authorities to improve rural sanitation?

Many a severe cold ends in tuberculosis?

Sedentary habits shorten life?

Neglected adenoids and defective teeth in childhood menace adult health?

A low infant mortality rate indicates high community intelligence?

